# Optometry in Nepalese Context: The Profession Beyond Providing Refraction Services

**DOI:** 10.31729/jnma.3523

**Published:** 2019-02-28

**Authors:** Dinesh Kaphle, Himal Kandel, Prakash Paudel

**Affiliations:** 1 School of Optometry and Vision Science, Queensland University of Technology, Brisbane, Australia; 2 Faculty of Medicine, Nursing and Health Sciences, Flinders University, Adelaide, Australia; 3 BPK Lions Centre for Ophthalmic Studies, Institute of Medicine, Kathmandu, Nepal

**Keywords:** *Nepal*, *optometry*, *refraction services*

## Abstract

Optometry is an independent profession which is specialised for providing comprehensive eye care including refraction and dispensing services, diagnosis and management of eye diseases and visual rehabilitation. In clinical settings of Nepal, optometrists are primarily recognised as refractionists and are provided with working opportunities in the same area. This report highlights other optomet-ric services such as binocular vision, multifocal lenses, contact lenses and occupational lens design which can be provided by optometrists besides performing refraction and prescribing spectacles. Considering large proportion of optometrists with further education and being working outside the country, new specialised services can be introduced through training and workshop to the fellow optometrists so that specialised services can reach up to the public level.

## INTRODUCTION

The World Council of Optometry (WCO) defines optometry as “A healthcare profession that is autonomous, educated, and regulated (licensed/ registered), and optometrists are the primary healthcare practitioners of the eye and visual system who provide comprehensive eye and vision care, which includes refraction and dispensing, detection/diagnosis and management of disease in the eye, and the rehabilitation of conditions of the visual system.“^1^ Whether or not the optometry profession in Nepal complies with the definition of the WCO is debatable. First, the profession is not autonomous and does not have own independent regulating body yet. Second, the optometrists are not well recognised as the primary eye care providers. In this article, we briefly discuss history of optometry education in Nepal, and explore the optometric speciality services that could be available besides existing refraction services.

## OPTOMETRY IN NEPAL

Optometry education began in Nepal in 1997 with the establishment of a Bachelor of Optometry programme at the Institute of Medicine, Tribhuvan University.^2^ Even after two decades of the establishment, the profession is still in its infancy stage. Public do not know what optometry is. Even many health care professionals are unaware of the scope of practice of optometry profession. There may be several reasons for a poor recognition of the internationally recognized profession in Nepal. There is an overlap of scope of practice between optometrists, ophthalmologists and ophthalmic assistants. Another reason for the poor recognition could be that optometrists are not currently providing full spectrum of speciality services such as low vision rehabilitation, occupational lens design largely due to lack of resources.^3^ In many settings, optometrist's role is limited to providing refraction services. The refraction services are provided by other eye professionals as well.^2^ Perhaps, providing specialized services would enable wider recognition of optometry as an independent profession among other health care professionals and general public. Some of the specialised optometric services, that highlight why the specialized optometry services are important in the Nepalese context, are discussed here.

### Binocular vision

The coordination of the two eyes is important not only for increasing the field of view but also for having stereoscopic vision (depth perception) and comfortable vision for tasks at near, intermediate and far. It is important to note that having normal vision (Snellen acuity 6/6) at distance in both eyes does not necessarily mean that your eyes are working in a coordinated manner. Symptoms like headache particularly in the afternoons, blurred vision after few minutes of near work, asthenopia (eye strain) may be related to binocular vision disorders and can be managed with special treatment methods such as prisms and bifocals.

### Prescribing Prisms

The prisms play an important role for the management of decompensated heterophoria (latent strabismus), tro-pias (manifest strabismus), esotropic diplopia, homonymous hemianopia and head tilt.^4^ It is important to note that not all patients may benefit from the prism therapy. The general guideline for prism prescribing is that patient has to be symptomatic before the treatment and the prism should not make binocular vision worse. Although optometrists are trained to weigh risks and benefits of prescribing prism therapy, and for accurate prescription of prisms, it is not commonly prescribed by optometrists in Nepal.

Patients with an associated phoria (latent ocular deviation with the corrected prism in situ) may benefit from prescribed prism.^5^ However, there are several important considerations to make while prescribing prisms. For example, if the associated phoria returns to the value measured before putting the prism, it is likely that there is a little clinical benefit of prescribing prisms. There are few guidelines for prescribing prisms such as Sheard's criterion that suggests vergence reserves to keep at least twice the amount of heterophoria.^5^

Furthermore, accommodative esophoria has to be ruled out in patients with uncorrected or undercorrected hyperopia. There are some contraindications for prism therapy. For example, if the exophoria is of divergence excess (larger exophoria in the distance than in the near), prisms have to avoided because the prism can induce esophoria at near, therefore, it may make the symptoms worse. The direction of the base of the prism should be opposite to the direction of eye deviation. Base-In for exo deviation and Base-Out for eso deviations are standard prescribing recommendations. It is important to note that prisms are generally the second line of treatment when the vision therapy fails or there is poor compliance with the therapy.

### Bifocal or multifocal lenses

Apart from the conventional use in presbyopia management, bifocals or multifocal lenses may be used for management of binocular vision disorders, such as decompensated esophoria, accommodative esotropia and intermittent or constant esotropia in children.^6^ Bifocals are also useful for children with convergence excess and accommodative insufficiency, however, they are not recommended if children are too young for near vision, for example reading and writing. Children with accommodative esotropia are usually hyperopes who accept plus lenses for distance and they may be sufficiently treated with single vision lenses of full cycloplegic refractive correction. Whereas if single vision lenses straightens the eye but leaves the eye with more than 10 prism dioptres and does not fuse at near, bifocals work better to reduce or eliminate esotropia. ^6^ Bifocals are also prescribed for children with no strabismus in cases like convergence excess, where patient has symptomatic near esophoria with inadequate divergence ability. Binocular fusion should not be compromised with the addition power prescribed in the bifocals.

The prescription of bifocals can be determined by near point refraction, accommodative function (accommodative lag determined by dynamic retinoscopy) as well as positive and negative relative accommodation tests. Although the arbitrary rule is to prescribe addition of +2.5 D or +3.0 D, it is preferable to prescribe customize power. Bifocals with addition which is set for Harmon distance: distance from the elbow to the knuckle of the middle finger, produces binocular alignment and fusion. It is important to adjust the height of the bifocals for children of different age. For preschool children, a flat-top 28mm bifocal is preferred keeping the height high (at mid-pupil until 5 years and then at the lower pupil border until about 8 years) so that the child uses the addition segment all the time for near work. ^6^ Bifocals have added advantage over multifocals that parents and teachers can monitor where the child is looking through for near tasks. For sports or rough play, children can wear polycarbonate sports goggles without an addition.

### Contact Lenses

Besides optical correction, contact lenses can be used for therapeutic, drug delivery and cosmetic purpose.^7,8^ Severe dry eye, keratitis, keratoconus, Stevens-Johnson syndrome are some of the conditions which can be treated better with contact lenses. While spherical soft contact lenses are the most common type of contact lenses for refractive correction, soft toric and rigid gas permeable are also alternatives for special need. Ocular surface including tear film, corneal diameter and curvature and lens fitting have to be assessed before prescribing contact lenses. The wearing and replacement schedule depends upon the type of the lens, lens material and their oxygen permeability properties. Optometrists are specialised in contact lenses prescribing as well as managing the complications related to the lens wear.

### Low vision rehabilitation

Low visual rehabilitation is a multidisciplinary approach where people from different background work together to improve person's visual need. The low vision team usually comprises of ophthalmologists, low-vision specialists who are often optometrists, occupational therapists, orientation and mobility specialists, social workers, and counsellors. When a client is referred to a low vision rehabilitation clinic, a low vision evaluation will be performed by an optometrist or ophthalmologist. The low vision specialist assesses whether the client get benefitted from optical devices- spectacle magnifiers, stand magnifiers, telescopes, video magnifiers and non-optical devices-large prints, adjustable lamps, absorptive sunglasses, typoscope (black plastic with a cut-out opening) aids. With advancement in the technology, electronic devices such as computers, smartphones and tablets come with visual impairment friendly version such as having features of talking books, large screen, and adjustable brightness. Other items such as watches, blood pressure cuffs and blood sugar machines are also available in talking modes.

Optometrists have basic foundation in optics in their degree and most of the rehabilitation approach for low vision clients is around optics such as adjusting glare and appropriate magnification. Therefore, in the context of Nepal it would be easier to use existing manpower, optometrists, for low vision rehabilitation services rather than training ophthalmologists or ophthalmic assistants specifically for that.

### Occupational Lens Design

Some occupations require personal protective equipment (PPE) for specific task execution. Trivex and polycarbonate lens material are used for high impact resistance safety spectacles or for children's spectacles.^9^ Polycarbonate have disadvantages of being scratched easily. Occupations like builders or wielders are recommended to wear safety spectacles-goggles and shields, which have lens material extended to the sides of the frame so that foreign objects are prevented to enter the eye and surrounding area. The lens material of safety spectacles is usually made up of plastic (thickened CR 39) or toughened glass (thermally or chemically), low energy impact materials or polycarbonate or Trivex.^9^

Ergonomics- study of ‘fit’ between people and their work, has to be considered before prescribing spectacles for occupational use. For example, anti-reflection coating spectacles are recommended for visual display unit (VDU) users along with consideration in the screen position, chair position, glare control, regular breaks in between, and illumination adjustment. Occupational progressive lenses (OPALs) are available of varying distance, intermediate and near zone diameters. For instance the design of the OPALs are different for patients who have primary purpose for computer work compare to those who deal mostly with customers like receptionists or do driving.

### New advancements in myopia control

Myopia, which is estimated to affect a half of the global population by 2050, is a very familiar condition among optometrists.^10^ However, are Nepalese optometrists up to date with new strategies for delaying the onset and control the progression to prevent visual impairment caused by high myopia. A free online myopia calculator, https://calculator.brienholdenvision.org/, developed by the Brien Holden Vision Institute, provides the best strategy available to treat the condition based on a child's age and preferred type of refractive correction. ^11^ Studies have found that bifocals help reduce the progression in children with decreased accommodative response.^12^ It is believed that children with reduced accommodative response have blur on peripheral retina, which stimulates the axial length elongation. Bifocals prevent myopia progression by reducing the blur on the peripheral retina. Progressive addition lenses, act similar like the bifocals for myopia control, but may require longer adaptation because of peripheral distortions.

Orthokeratology, wearing contact lenses in the night to have clear vision for the day, is an alternative management of refractive errors. It would be interesting to know how many of Nepalese optometrists are eager to introduce new management strategies in their clinical practice.

### Challenges and the Way Forward for Expanding Optometry Services

Since the inception of optometry programme in Nepal, the growth in optometry is minimal due to several challenges. It is accepted that when the optometry course was started there were no sub-specialised optometrists in the country. Although the core optometric procedures such as prisms and paediatric bifocals are in the existing curriculum, they are not extensively practiced during study and hardly used in clinical practice afterwards. However, the situation is different now. Nearly 60% of the Institute of Medicine graduates have obtained masters or PhD degree and quite a few are practicing optometry in developed countries.^13^ It should be possible to share the knowledge and skills from specialised optometrists, who may be either already in the country or while visiting the country for short period, to broader general optometrists through Continuing Professional Development (CPD). Nepalese association of Optometrists (NAO) could initiate specific CPDs to general optometrists so that new techniques can be gradually introduced in practice. It is expected that in the early phase, the practitioners may feel hesitated about changing their traditional way of management. But it has to be introduced sooner than later to provide the best tertiary eye services particularly in the field of binocular vision, myopia, and occupational optometry to the public. Patients can be reassured that a new management strategy is being introduced and their feedback about the treatment would be helpful to improve the current treatment plans. However, patients should not imposed with extra financial burden for introducing new treatment strategies. For example, if an optometrist is prescribing a prism to treat decompensated phoria which was not responding to vision therapy, the first pair of spectacles with prisms can be provided either free or on subsidized price.

## CONCLUSIONS

Although Nepal Health Professional Council (NHPC) is supposed to regulate optometry profession in the country, there is no representative in its board members from optometrists. It would be very difficult to bring any regulating optometry laws through the NHPC unless optometry have their own optometry council.

In conclusion, optometrists, although independent professional, should work in collaboration with other eye and health professionals towards achieving a common goal: providing better eye care services to everyone. Working opportunities must be provided so that optometrists will be encouraged to serve within the country. Specialised optometric services, besides refraction, can be provided to the public if the knowledge and skills are transferred from the trained personnel to the fellow optometrists through trainings and workshops. The professional body, the NAO, should take responsibility to introduce specialised optometric services so that the standard of eye care would be optimum for the general public.

## Conflict of Interest

**None.**

## References

[1] World Council of Optometry Who is an optometrist?.

[2] Kandel H, Murthy G, Bascaran C. (2015). Human resources for refraction services in Central Nepal. Clin Exp Optom..

[3] Kandel H. (2013). Situational Analysis of Refractive Error Services in the Central Region of Nepal. London: University of London;.

[4] American Academy of Ophthalmology (2018). Prescribing prism [Internet].

[5] Gray L. (2008). The prescribing of prisms in clinical practice. Graefes Arch Clin Exp Ophthalmol..

[6] Healio (2002). Bifocals in kids: Thorofare, [Internet].

[7] Denaeyer GW. (2010). Therapeutic applications of contact lenses. Contact Lens Spectrum..

[8] Wilson CG. (2004). Topical drug delivery in the eye. Exp Eye Res..

[9] Association of British Dispensing Opticians (2017). Occupational dispensing- A reference guide to professional advice and solutions for the workplace [Internet].

[10] Holden BA, Fricke TR, Wilson DA, Jong M, Naidoo KS, Sankaridurg P (2016). Ophthalmology.

[11] Brien Holden Vision Institute (2017). Myopia calculator Sydney [Internet].

[12] Review of Optometry (2012). Slowing myopia progression in children Pennsylvania, [Internet].

[13] Kaphle D, Marasini S. (2018). Seeing clearly Kathmandu [Internet]. Kantipur Publications;.

